# An Enhanced Robust Control Algorithm Based on CNF and ISM for the MEMS Micromirror against Input Saturation and Disturbance

**DOI:** 10.3390/mi8110326

**Published:** 2017-11-03

**Authors:** Jiazheng Tan, Weijie Sun, John T. W. Yeow

**Affiliations:** 1 College of Automation Science and Engineering, South China University of Technology, Guangzhou 510000, China; tanjiazheng1992@outlook.com (J.T.); auwjsun@scut.edu.cn (W.S.); 2 Advanced Micro-/Nano-Devices Lab, Department of Systems Design Engineering, Waterloo Institute for Nanotechnology, University of Waterloo, 200 University Avenue West, Waterloo, ON N2L 3G1, Canada

**Keywords:** micromirror, input saturation, disturbance rejection, composite nonlinear feedback, integral sliding mode

## Abstract

Input saturation is a widespread phenomenon in the field of instrumentation, and is harmful to performance and robustness. In this paper, a control design framework based on composite nonlinear feedback (CNF) and integral sliding mode (ISM) technique is proposed for a MEMS micromirror to improve its performance under input saturation. To make the framework more effective, some essential improvements are supplied. With the application of the proposed design framework, the micromirror under input saturation and time-varying disturbances can achieve precise positioning with satisfactory transient performance compared with the open-loop performance.

## 1. Introduction

Since the first micromirror for scanning applications was reported in 1980 [1], the research of microelectromechanical systems (MEMS) micromirrors has become increasingly popular. Early MEMS micromirrors focused on imaging applications such as confocal microscopy [2] and fingerprint sensing [3]. Later, it was extended to some other different areas such as optical coherence topography [4], optical switches [5], and high-resolution displays [6]. Recently, some new applications include response surface method [7] and ghost imaging [8]. On the basis of actuation method, torsional micromirrors can be classified into different types: electrothermal [9], electrostatic [10], electromagnetic [11,12], piezoelectric [13], and so forth. Recently, the electromagnetic micromirrors have attracted special attention owing to their ability to produce large scan angles with low voltage and remote actuation (meaning that the mirrors can be controlled by the non-contact force at a distance).

**Figure 1:**
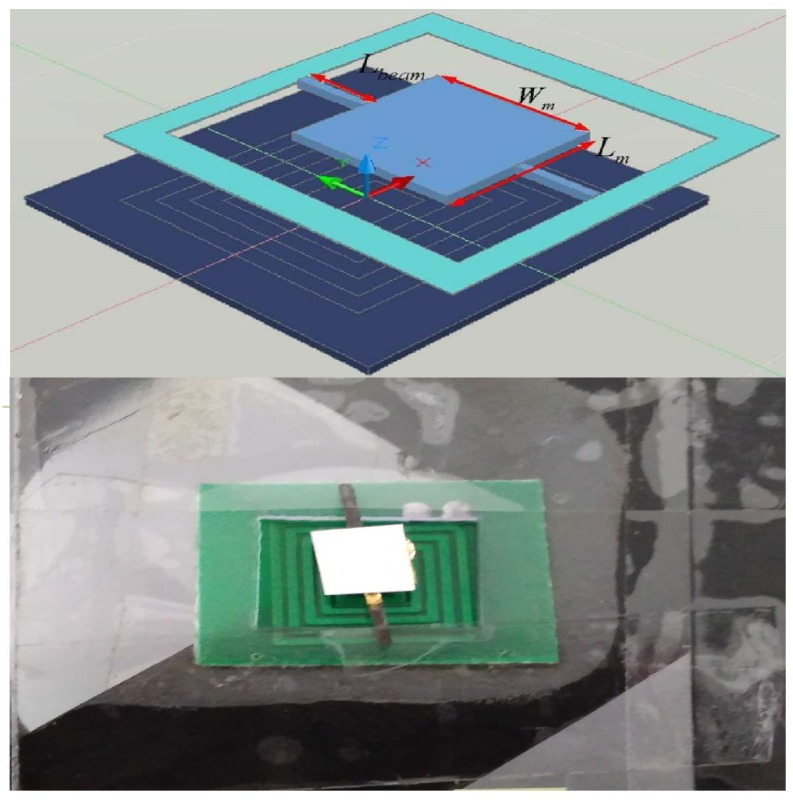
The structure of the micromirror. The micromirror consists of the micromirror-plate, the torsional beam, the driving microcoil, a support structure and other parts. Two straight torsion micromirror bars are fixed on the frame at a distance position 1 mm from the left side center of the mirror to coil center. The axis of rotation of the scanner is along *Y* vector. Rectangular spiral coil are assembled to the bottom of the frame in the *X*, *Y* plane and the center of the coil is the origin in Cartesian Coordinate.

In this paper, we study the electromagnetic micromirror reported in [11]. which was made by Pallapa and Yeow. The structure of the mirror is shown in Figure 1. In their original work, it is reported that the mirror was fabricated by the application of hybrid MEMS fabrication using polymers and magnets [11]. In the following the fabrication process is briefly provided [6]. In the first place, adopting the lithography method, the mould of the micromirror and two torsional bars are fabricated with a silicon wafer used as the mould substrate. The mould is fabricated by standard photolithography. Next, polydimethylsiloxane (PDMS) is filled into the mould as a basement of the micromirror and two torsional bars, and the MQFP-12-5 isotropic magnetic powder (Nd-Fe-B) (Magnequench International Inc., Pendleton, IN, USA) with a particle size D50 of 5 μm is doped into the polydimethylsiloxane (PDMS) at the weight percentage of 80%. Subsequently, an ultrasonic horn tip probe is immersed into the composite, therefore leading to uniform dispersion. Then, using the e-beam evaporation approach, the micromirror is plated with a layer of gold with a thickness of 1.0 mm as a reflective layer. Finally, the micromirrors are magnetized under a field of 1.8 Tesla, resulting in that the micromirror contains a hard magnetic feature. Besides, the rectangle coils are fabricated by the standard printed circuit board (PCB) manufacturing technique and constructed under the micromirror. The detailed design parameters of the mirrorare shown in Table 1.

To rotate an electromagnetic micromirror, a peripheral driving circuit based on a microcoil is essential. Clearly, the amplitude of the driving circuit’s output is limited, meaning that the plant’s input is subject to input saturation. Therefore, it is essential to propose a suitable method to guarantee the performance of the micromirror under input saturation. Because the mathematical model of the micromirror is linear [12], we will search for a solution to the input saturation problem in the field of linear system control theory.

The input saturation problem has been extensively investigated in the field of linear system control theory. For this problem, transient performance of the closed-loop system should not be neglected. Settling time and overshoot are the two most important evaluation indices of transient performance. However, it is unfortunate that short settling time and negligible overshoot are usually contradictory aspects when facing input saturation, which means that most of the existing control techniques cannot help but make a trade-off between them. As a kind of nonlinear control scheme, the composite nonlinear feedback (CNF) technique has been presented to tackle such a dilemma [14]. The CNF technique is composed of two components; i.e., a linear part and a nonlinear part. The linear feedback part is introduced to yield a closed-loop system with small damping ratio, while the nonlinear feedback part is designed to increase the damping ratio when the output signal reaches the reference asymptotically. Therefore, it is possible for the CNF technique to simultaneously achieve quick response and negligible overshoot. The CNF technique has also been implemented in various of engineering applications such as hard disk drive servo systems [15,16,17], helicopter flight control systems [18,19], position servo systems [20,21], and grid-connected voltage source inverters [22].

The normal CNF technique is able to improve the transient performance of controlled systems in the absence of external disturbances [15,16]. However, disturbances are non-negligible in practical environments, especially for the problems in high-precision control fields. Thus, both the enhanced composite nonlinear feedback and robust composite nonlinear feedback techniques are formulated to deal with constant disturbances [17,21]. Without considering input saturation, robust design for CNF technique has also been achieved by improving the nonlinear part to deal with unmodelled dynamic effects [23]. Furthermore, due to the easy implementation property, the integral sliding mode technique has been combined with the CNF technique for the rejection of time-varying disturbances under input saturation [24,25].

**Figure 2:**
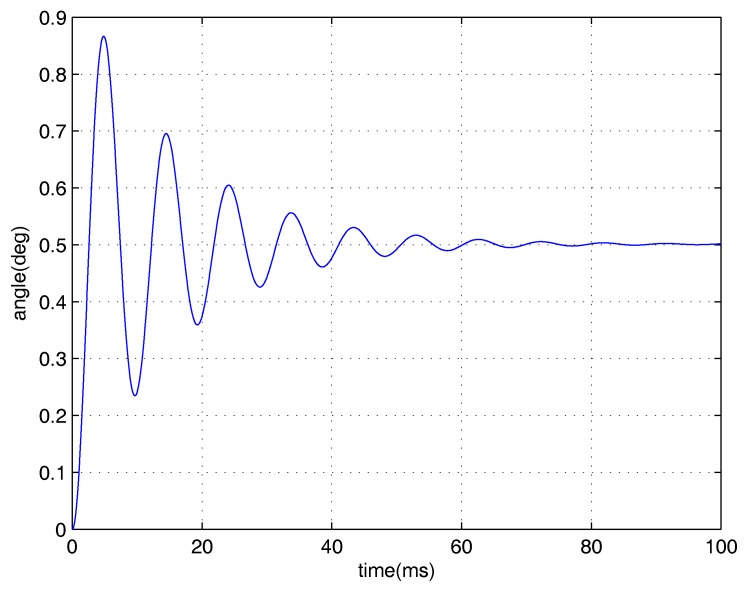
Open-loop positioning performance under input saturation (Theoretical model simulation).

Our motivation is to achieve good transient performance and disturbance rejection for the micromirror simultaneously under input saturation. Previous work of control system design for the electromagnetic micromirror can be seen in [26,27]. However, input saturation was not considered in those works. As shown in Figure 2, the open-loop performance of the micromirror is far from favourable (long settling time and large overshoot), meaning that further improvement is essential (the difference between the two responses is mainly because of the unmodelled dynamics and the air damping effect). Inspired by the aforementioned discussion, in this paper, we apply the integral sliding mode (ISM)-based CNF design framework considered by Bandyopadhyay, Deepak, and Kim [25]. Furthermore, to improve this framework, some improvements are introduced. With the proposed improvement, it is expected that the proposed control scheme would have much better robustness under input saturation than the existing work [25], while the transient and steady-state performance become much more satisfying. The effectiveness of the proposed design framework is illustrated by experimental results, and it would show that the proposed framework forces the micromirror to perform well under input saturation and disturbance.

This paper is organized as follows. In Section 2, the whole control design framework and the relevant improvements are proposed. Then, the effectiveness of the enhanced control scheme is examined by experimental results in Section 3. In Section 4, conclusions are drawn.

## 2. ISM-Based CNF Control Design

Consider a linear plant described in the following form with input saturation and disturbance:

(1) $$\dot{x}=Ax+Bsat\left(u\right)+Bw\left(x,t\right),x\left(0\right)=x_{0}\;y=Cx$$

where $x\in R^{n},\;u\in R,\;y\in R$, and w∈R are the state, control input, output, and disturbance input of the system. A,B,C are constant matrices with appropriate dimensions.

The function, sat(·):R→R, represents the input saturation defined as

(2) $$sat\left(u\right)=sgn\left(u\right)min\{u_{max},|u|\}$$

where $u_{max}$ is the saturation level of the input.

To introduce the whole control design framework, the following standard assumptions on the given system are made

A1: (A,B) is stabilizable.

A2: (A,C) is detectable.

A3: (A,B,C) is invertible with no invariant zero at s=0.

A4: w(x,t) represents the bounded matched uncertainty or disturbance and

(3) $$\left|w\left(x,t\right)\right|\leq w_{max}$$

where $w_{max}$ is the maximum amplitude of *w*.

In the following, we will introduce an ISM-based CNF scheme for system (Equation (2)) such that the controlled output *y* can asymptotically achieve the positioning of reference input *r* under constant or time-varying disturbances within limits.

The ISM-based CNF scheme can be constructed by the following a step-by-step design procedure.

(1) Design the part of CNF control law [17].

The linear feedback part is first given as

(4) $$u_{L}=Fx+Gr$$

*r* denotes the constant reference signal, *F* is the feedback gain of the states, and *G* is the feedforward gain of the reference. *F* is chosen such that (A+BF) is a Hurwitz matrix and the closed-loop system $C{\left(sI-A+BF\right)}^{-1}B$ has a small damping ratio [15].

Besides, *G* is defined as

(5) $$G=-{\left[C{\left(A+BF\right)}^{-1}B\right]}^{-1}$$

Next, the nonlinear feedback part is formulated as

(6) $$u_{N}=\rho \left(y-r\right)B^{T}P\left(x-x_{e}\right)$$

where

(7) $$x_{e}=-{\left(A+BF\right)}^{-1}BGr$$

and ρ(y−r) is a non-positive nonlinear function locally Lipschitz in (y−r). $u_{N}$ is introduced to change the system closed-loop damping ratio as the output approaches the step command input [15]. In this paper, we select

(8) $$\rho \left(y-r\right)=-\beta e^{-\alpha \alpha_{0}\left|y-r\right|}$$

where α and β are positive parameters to be tuned, and

(9) $$\alpha_{0}=\left\{\begin{array}{cc} \frac{1}{|h_{0}-r|}, & r\neq h_{0}\\ 1, & r=h_{0} \end{array}\right.$$

For simplicity, we set $h_{0}=0$ in this paper. With the discontinuous coefficient $\alpha_{0}$, ρ(y−r) can suit the amplitude variation of constant reference [16].

In (6), P>0 is the solution to the following Lyapunov equation:

(10) $${\left(A+BF\right)}^{T}P+P\left(A+BF\right)=-W$$

where *W* is a positive-definite matrix. It is known that the solution *P* always exists if (A+BF) is Hurwitz.

For convenience, we set *W* as

(11) $$W=10^{\theta }\cdot \hat{E}$$

where $\hat{E}$ represents the appropriate dimensional identity matrix and θ∈R.

In this respect, the linear feedback part and nonlinear feedback part can be combined to form the CNF control law

(12) $$u_{CNF}=u_{L}+u_{N}=Fx+Gr+\rho \left(y-r\right)B^{T}P\left(x-x_{e}\right)$$

The following theorem shows that the closed-loop system comprising the given plant (Equation (2)) with and the CNF control law of Equation (12) is asymptotically stable. It also determines the magnitude of *r* that can be tracked by such a control law without exceeding the control limit [17].

**Theorem** **1.** *Under assumptions A1–A4, for the closed-loop system composed of Equations* (2) *and* (12)*, if the following conditions are satisfied*

*(1) For any δ∈(0,1), there exists a largest positive scalar $c_{\delta }>0$ such that*

(13) $$\forall \tilde{x}\in X\left(F,c_{\delta }\right):=\left\{\tilde{x}:{\tilde{x}}^{T}P\tilde{x}\leq c_{\delta }\right\}\Rightarrow \left|F\tilde{x}\right|\leq \left(1-\delta \right)u_{max},$$

*where 0<δ<1 and*

(14) $$\tilde{x}=x-x_{e}$$

*(2) The initial condition $x_{0}$ satisfies*

(15) $$x_{0}-x_{e}\in X\left(F,c_{\delta }\right)$$

*(3) The amplitude of the reference satisfies*

(16) $$\left|Hr\right|\leq \delta_{1}u_{max}$$

*where $0<\delta_{1}<\delta$, $H=FG_{e}+G$.*

*Then, for any non-positive function ρ(y−r) locally Lipschitz in (y−r), the control law (Equation* (12)*) can stabilize the system (Equation* (2)*) and drive the output of Equation* (2) *to asymptotically track the command reference in the absence of disturbances w as well as in the presence of input saturation [15].*

(2) Design the part of integral sliding mode law.

Define the following notations:

(17) $$x_{d}=\int_{0}^{t}\left(Ax\left(\tau \right)+Bu_{CNF}\left(\tau \right)\right)d\tau \;e=C_{0}\left(x-x_{d}\right)$$

where $C_{0}$ is an appropriate matrix satisfying $C_{0}B>0$.

$x_{d}$ represents the trajectory of the nominal plant (the original plant that does not suffer from disturbance). It is clear that the boundedness of $x_{d}$ is equivalent to the stability of the closed-loop system (Equation (2)) with the CNF control law (Equation (12)) in the absence of disturbances *w* and input saturation, and it has been proved in [17].

The origin of *e* is the existence of *w*, and the goal of the integral sliding mode law is to eliminate the influence of *w*, forcing *x* to follow $x_{d}$.

Further, choose the following sliding manifold

(18) $$s\left(e,t\right)=k_{1}e+k_{2}\int_{0}^{t}e\left(\tau \right)d\tau$$

where $k_{1}$, $k_{2}$ should be properly chosen to ensure $\dot{s}\left(e,t\right)=0$ is strictly Hurwitz.

Different from the work of Bandyopadhyay, Deepak, and Kim [25], an additional integral term $\int_{0}^{t}e\left(\tau \right)d\tau$ is supplied to the sliding manifold (Equation (18)). The effect of the integral term is twofold. Firstly, it can bring to faster responses, which is also proved by [28]. Furthermore, it can better force the trajectory to stay on the sliding surface *s* = 0 in the presence of parameter perturbation (because it is made of polymer, the mirror is soft, meaning that it is likely for it to suffer from parameter perturbation).

Construct the following Lyapunov function:

(19) $$V=\frac{1}{2}{s(e,t)}^{T}s\left(e,t\right)$$

It can be calculated that

(20) $$\begin{array}{cc} \dot{V} & =\;\left[k_{1}C_{0}\left(\dot{x}-{\dot{x}}_{d}\right)+k_{2}C_{0}\left(x-x_{d}\right)\right]s\left(e,t\right)\\ & =\;\left\{k_{1}\left(u_{ISM}+w\right)+k_{2}\int_{0}^{t}\left[u_{ISM}\left(\tau \right)+w\left(\tau \right)\right]d\tau \right\}C_{0}Bs\left(e,t\right) \end{array}$$

Accordingly, the integral sliding mode control law is constructed as

(21) $$u_{ISM}=u_{eq}+u_{sw}$$

where $u_{eq}$ is the equivalent control and $u_{sw}$ is the switched control.

Let

(22) $$u_{eq}=-w\left(x,t\right)$$

and

(23) $$u_{sw}=-Msign\left[C_{0}Bs\left(e,t\right)\right]-N\left[C_{0}Bs\left(e,t\right)\right]$$

where M,N are positive parameters and $M\geq w_{max}$.

The term $-Msign[C_{0}Bs\left(e,t\right)]$ is discontinuous, and it probably causes high-frequency unmodeled dynamics. The high-frequency unmodeled dynamics can lead to the chattering phenomenon, which seriously harms the actuator and the direct application of the mirror. The application of the continuous term can reduce the discontinuous effect. Therefore, the term $-N[C_{0}Bs\left(e,t\right)]$ is introduced to reduce chattering, which is different from the work of Bandyopadhyay, Deepak, and Kim [25].

Applying the ISM law (Equation (21)), it is obvious that

$$\dot{V}\leq 0,\;\left(\dot{V}=0,s=0\right)$$

Thus

$$\lim_{t\to \propto }e=0\Rightarrow \lim_{t\to \propto }x\left(t\right)=x_{d}\left(t\right)$$

So

(24) $$\lim_{t\to \propto }y\left(t\right)=\lim_{t\to \propto }Cx\left(t\right)=Cx_{e}=-C{\left(A+BF_{x}\right)}^{-1}BGr=r$$

Now, the whole control scheme composed of (Equations (12) and (21)) could be expressed as

(25) $$u=u_{CNF}+u_{ISM}$$

and it can stabilize the system (Equation (2)) and drive the output of Equation (2) to asymptotically track the command reference under the above conditions (Equations (13), (15) and (16) ) in the presence of disturbances *w*.

When we design the ISM part, the input saturation is not taken into account. Clearly, to ensure that the amplitude of Equation (25) will not go beyond the limit, the constraint of the maximum amplitude of *w* is essential.

Therefore, we need the following theorem.

**Theorem** **2.** *Under assumptions A1–A4 and the conditions Equations* (13)*,* (15) *and* (16)*, for the closed-loop system composed of Equations* (2) *and* (25)*, if the maximum amplitude of disturbance satisfies*

(26) $$|\left(\delta -\delta_{1}\right)u_{max}|=w_{max}$$

*then, by the application of Equation* (25)*, the output of Equation* (2) *can asymptotically track the command reference and the amplitude of Equation* (25) *will not go beyond the limit [25].*

**Figure 3:**
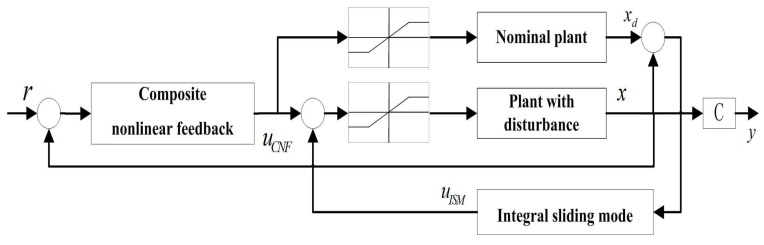
The integral sliding mode-composite nonlinear feedback (ISM-CNF) controller diagram.

The block diagram of the proposed control design is shown in Figure 3.

## 3. Experimental Results

**Figure 4:**
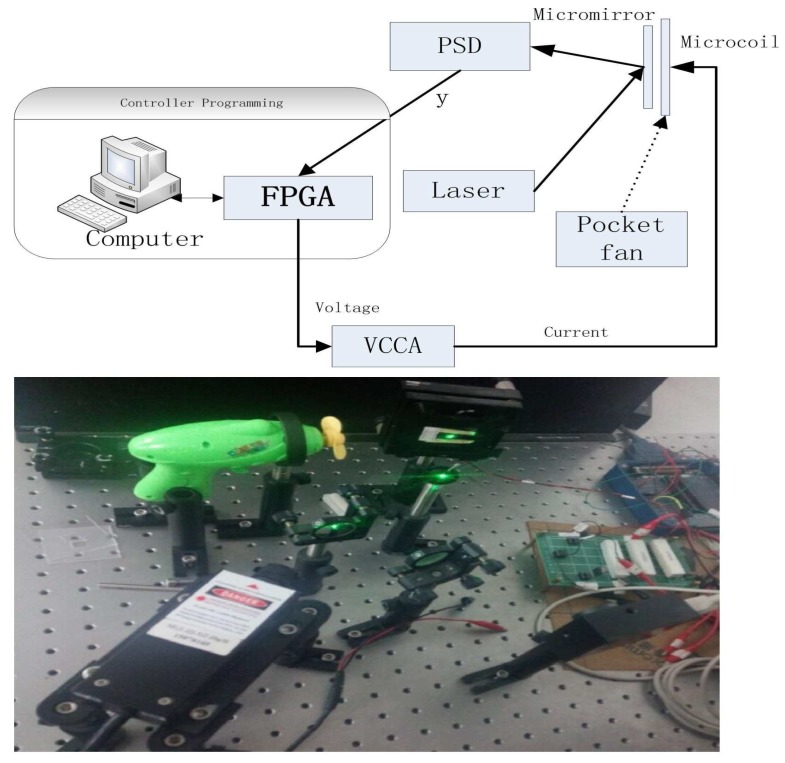
The experimental platform. FPGA: field-programmable gate array; PSD: position-sensitive detector; VCCA: voltage-controlled current amplifier.

In this section, we validate the performance of the aforementioned control scheme composed of composite nonlinear feedback and integral sliding mode technique. The validation is performed on a torsional micromirror-based experimental platform. The platform is composed of a He-Ne laser, a micromirror together with coils, a PSM2-10 position-sensitive detector (PSD), a voltage-controlled current amplifier (VCCA) circuit, and an NI PXI-7852R field-programmable gate array (FPGA) system (see Figure 4). The structure of the micromirror is depicted in Figure 1. The controller is programmed in the FPGA card. The output of the FPGA card, which is the output of the controller, is in the form of voltage. The voltage signal is converted to current by the VCCA circuit proportionally, and the ratio is one to one. A magnetic field sets up when the currents are flowing in the coils, resulting the Lorentz force to rotate the mirror. The amplitude of current is restricted within 1.0 A because the coils have a restriction on maximum passing current.

**Figure 5:**
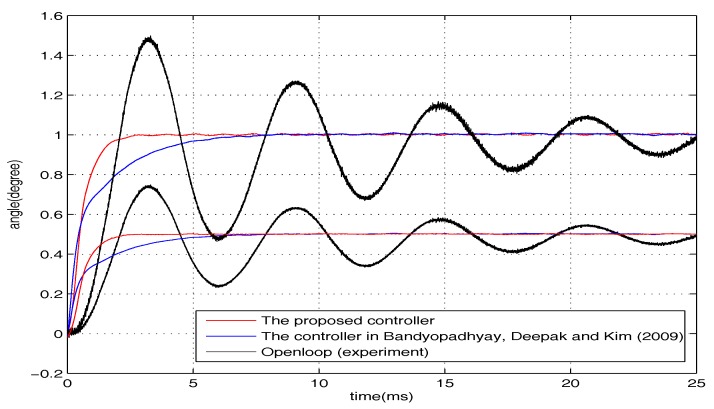
Positioning performance under input saturation.

The model of the micromirror under external disturbance and input saturation is shown as follows [12]

(27) $$\left[\begin{array}{c} {\dot{x}}_{1}\\ {\dot{x}}_{2} \end{array}\right]=\left[\begin{array}{cc} 0 & 1\\ -\frac{k}{J} & -\frac{b}{J} \end{array}\right]\left[\begin{array}{c} x_{1}\\ x_{2} \end{array}\right]+\left[\begin{array}{c} 0\\ \frac{1}{J} \end{array}\right]\left(sat\left(u\right)+w\right)\;\hat{y}=\left[\begin{array}{cc} 1 & 0 \end{array}\right]\left[\begin{array}{c} x_{1}\\ x_{2} \end{array}\right]=x_{1}$$

where $x_{1},x_{2},u$ are respectively the angular position of the micromirror, the angular velocity, and the driving torque. $J=6.26\times 10^{-12}\left(\mathrm{kg}\cdot m^{2}\right)$ is the moment of inertia, $b=0.82\times 10^{-9}\left(N\cdot m\cdot s\right)$ is the damping coefficient, and $k=2.96\times 10^{-6}\left(N\cdot m\right)$ is the spring coefficient of the torsional bars. The practical open-loop performance is shown in Figure 5. One may have noticed some differences between the theoretical open-loop performance and practical open-loop performance (settling time and overshoot). The possible reasons are shown as follows. Firstly, the mirror’s practical model is not totally linear. In other words, the nonlinear unmodelled dynamics can influence the performance. Secondly, in the theoretical model of the mirror, air frication is not considered, which can lead to smaller overshoot and shorter settling time.

The controller parameters are chosen as follows. For the CNF part, $F=[-0.58\;-6.2\times 10^{-4}]$, $\alpha =8.7\times 10^{-5}$, $\beta =2.6\times 10^{-5}$, θ=0.268. For the ISM part, we select M=1, N=2.5, $k_{1}=450$, $k_{2}=2$, $C_{0}=\left[1\;J\right]$. To summarize, the proposed scheme is achieved as the sum of two parts.

In the following experiment, to state the results more clearly, we also provide the performance of the controller proposed by Bandyopadhyay, Deepak, and Kim. The controller is shown as follows:

(28) $$\begin{array}{ccc} u_{BDK} & = & u_{CNF}+{\bar{u}}_{ISM}\\ {\bar{u}}_{ISM} & = & u_{eq}+{\bar{u}}_{sw}\\ {\bar{u}}_{sw} & = & -\bar{M}sign\left[\bar{G}B\bar{s}\left(e,t\right)\right]\\ \bar{s} & = & \bar{G}\left(x-x_{d}\right) \end{array}$$

Obviously, the difference between the two controllers is in the part of ISM. Therefore, in the part of CNF, we select the same parameters. In the part of ISM, for the above controller, we select $\bar{G}=\left[0\;J\right]$, $\bar{M}=500$.

The positioning experimental results are shown in Figure 5, in which the output responses of the closed-loop system and open-loop system are given. The figure has shown that the torsional micromirror can achieve the expected precise positioning with the distinguished features (i.e., high-speed seeking performance and negligible overshoot under control input saturation). Clearly, the positioning performance of the proposed control scheme under input saturation is validated.

**Figure 6:**
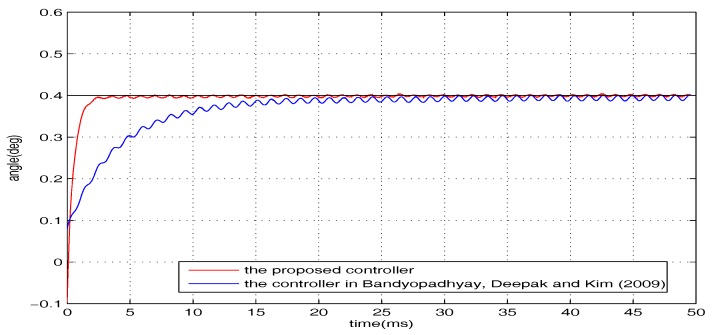
*r* = $0.4^{\circ }$, $w=w_{1}$.

**Figure 7:**
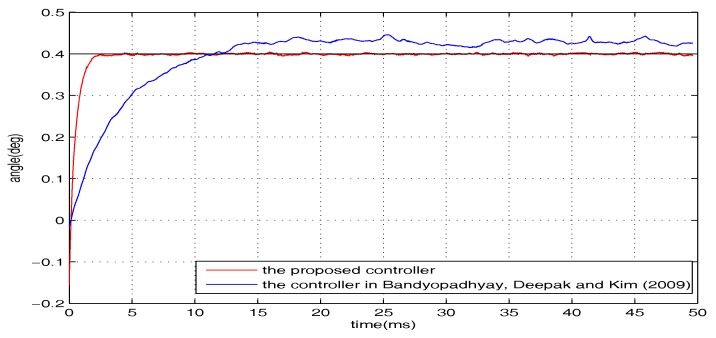
*r* = $0.4^{\circ }$, $w=w_{2}$.

**Figure 8:**
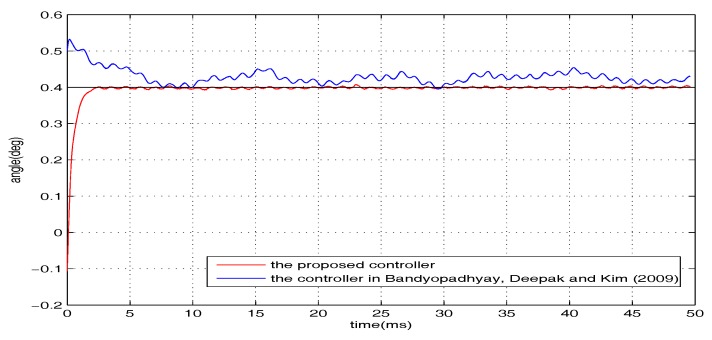
*r* = $0.4^{\circ }$, $w=w_{1}+w_{2}$.

To examine the robustness, additional introduced disturbances are imposed on the closed-loop system. The whole disturbances are divided into two categories (i.e., $w_{1}$ and $w_{2}$, where $w_{1}=0.3$sin(1200t) V is presented in the form of voltage and $w_{2}$ is the wind interference generated by a pocket fan, as shown in Figure 4). The level of the disturbance from the pocket fan is equivalent to a voltage disturbance with the amplitude of 0.34 V. The regulation performance of the torsional micromirror under the following three cases are studied: (1) *r* = $0.4^{\circ }$, $w=w_{1}$; (2) *r* = $0.4^{\circ }$, $w=w_{2}$; (3) *r* = $0.4^{\circ }$, $w=w_{1}+w_{2}$. The experimental results are shown in Figure 6, Figure 7 and Figure 8. Compared with the controller proposed by Bandyopadhyay, Deepak, and Kim [25], the proposed scheme with the improvement forces the outputs to settle into the target asymptotically. It indicates that the whole design framework is workable and the proposed improvement is essential.

In general, the overall results state clearly that the proposed scheme is effective in improving the transient and steady-state performance and robustness under input saturation.

## 4. Conclusions

In this paper, to deal with input saturation and achieve disturbance rejection for the MEMS micromirror, a robust control design framework based on composite nonlinear feedback and integral sliding mode is proposed, and some essential improvement is supplied to further enhance the framework. The effectiveness of the proposed scheme is verified by experimental results. Applying the proposed scheme, the micromirror can deal with input saturation and time-varying disturbances at the same time, while providing much better transient and steady-state performance compared with the open-loop performance.

## Figures and Tables

**Table 1:** Paramenters of the micromirror.

Symbol	Parameter	Value
$W_{beam}$	width of the torsion bar	250 μm
$L_{beam}$	length of the torsion bar	2 mm
$t_{beam}$	thickness of the torsion bar	250 μm
$W_{m}$	width of the mirror	4 mm
$L_{m}$	length of the mirror	4 mm
$t_{m}$	thickness of the mirror	250 μm
